# Renal Dose-Adjustment Concordance in Critically Ill Patients with AKI and CKD: A Cross-sectional Study

**DOI:** 10.5812/ijpr-172540

**Published:** 2026-10-06

**Authors:** Mahdi Amirzadeh, Zahra Sahraei, Shahed Ahmadi

**Affiliations:** 1 Faculty of Pharmacy, Shahid Beheshti University of Medical Sciences, Tehran, Iran; 2 Department of Clinical Pharmacy, School of Pharmacy, Shahid Beheshti University of Medical Sciences, Tehran, Iran

**Keywords:** Acute Kidney Injury, Chronic Kidney Disease, Renal Dose Adjustment, Dosing Discordance, Intensive Care Unit, Creatinine Clearance

## Abstract

**Background:**

Kidney dysfunction complicates drug dosing in the intensive care unit (ICU). Guidance is well established for stable chronic kidney disease (CKD) but is less certain in acute kidney injury (AKI), in which renal function can change rapidly.

**Objectives:**

To assess concordance between prescribed maintenance regimens and reference-based renal dose-adjustment recommendations for adults with AKI, CKD, or AKI superimposed on CKD.

**Methods:**

This cross-sectional study included 80 adults admitted to four ICUs in 2024. Acute kidney injury was classified according to the Risk, Injury, Failure, Loss, and end-stage renal disease (RIFLE) criteria using serial creatinine measurements and/or urine output, whereas CKD required documented chronicity. Cockcroft-Gault creatinine clearance (CrCl) was used to match patients to dosing-reference categories. Each patient–medication pair was classified once according to maintenance-dose amount and dosing interval, using UpToDate as the primary reference and the Sanford Guide as a supplementary source.

**Results:**

Among 414 patient–medication pairs, 211 (51.0%; patient-level cluster-bootstrap 95% CI, 46.2%–55.8%) were discordant: 125 were lower and 86 were higher than recommended. Vancomycin (41.4%), ciprofloxacin (40.0%), and levofloxacin (40.0%) had the highest drug-specific discordance proportions. Nephrology and pulmonology had the highest patient-level service proportions (62.5% and 61.5%, respectively).

**Conclusions:**

Reference discordance was frequent, particularly for antimicrobials. Because measured exposure and clinical outcomes were unavailable, these findings identify regimens requiring review rather than confirming prescribing errors.

## 1. Background

Kidney dysfunction can alter drug clearance, distribution, and exposure in critically ill patients, making individualized dosing important for safety and effectiveness (1-3). Acute kidney injury (AKI) is common in intensive care, chronic kidney disease (CKD) is frequently present at baseline, and CKD increases susceptibility to superimposed AKI and delayed recovery (3-5). These phenotypes differ in chronicity and renal function stability and should not be treated as interchangeable.

In relatively stable CKD, drug-information resources commonly provide dose or interval recommendations based on renal function assessed by creatinine clearance or estimated glomerular filtration rate (6). AKI is a non-steady-state condition in which serum creatinine may lag behind abrupt changes in kidney function; conventional equations may therefore misrepresent current drug clearance, and discordance with a reference table alone cannot establish inappropriate exposure (2, 7, 8). Nevertheless, recent hospital-based studies have reported frequent discordance with renal dose-adjustment guidance among patients with kidney impairment (9, 10). Both lower and higher exposures may be clinically important: insufficient exposure can compromise treatment, whereas accumulation can increase adverse effects. A transparent assessment must therefore specify the renal estimate, reference source, prescription timing, dose amount, and dosing interval.

Medication-safety studies use different terms, denominators, and reference standards, which can change the apparent frequency of discordance. A prescription-level analysis may count several medications for one patient, whereas a patient-level analysis records only whether any discordance occurred. Reference sources may also differ in their renal categories, acceptable dose ranges, and treatment indications. Therefore, a reproducible study must define the unit of analysis and the hierarchy used when recommendations conflict. In the present study, discordance was treated as a screening signal for further clinical review rather than a definitive diagnosis of medication error.

The Risk, Injury, Failure, Loss, and end-stage renal disease (RIFLE) framework classifies AKI into the severity categories Risk, Injury, and Failure and the outcome categories Loss and end-stage renal disease (ESRD). Kidney Disease: Improving Global Outcomes (KDIGO) provides the contemporary consensus framework for AKI diagnosis and staging and for CKD definition and classification (6, 11). Neither system is a direct drug-dosing algorithm for dynamic AKI. Intensive care unit (ICU) dosing is further complicated by fluid shifts, vasopressor use, augmented renal clearance, organ support, and rapidly changing treatment goals (1, 2, 10). A single creatinine-based estimate may therefore misclassify current drug clearance when kidney function is evolving.

## 2. Objectives

This study assessed concordance between prescribed maintenance regimens and predefined renal dosing recommendations in ICU patients with AKI, CKD, or AKI superimposed on CKD. We quantified overall and medication-specific discordance, differentiated lower- from higher-than-recommended regimens, and described prescribing-service patterns while avoiding causal inferences about individual prescribers or clinical drug exposure.

## 3. Methods

### 3.1. Study Design, Setting, and Ethics

This cross-sectional study was conducted from March to December 2024 in ICUs 3, 4, 5, and 8 at Loghman Hakim Hospital, a tertiary referral center affiliated with Shahid Beheshti University of Medical Sciences, Tehran, Iran. The study evaluated renal dose adjustment of medications prescribed by treating teams; antimicrobial selection, microbiological adequacy, and infection severity were not predefined outcomes. The protocol was approved by the institutional ethics committee (IR.SBMU.PHARMACY.REC.1403.159). Because the study was noninterventional, involved no direct patient contact, and used coded clinical-record data, written informed consent was waived and confidentiality was maintained.

### 3.2. Participants

Adults aged at least 18 years were eligible if they were admitted to a participating ICU, had AKI, CKD, or AKI superimposed on CKD, and received at least one medication requiring renal dosing consideration. AKI was identified and classified using RIFLE based on serial serum creatinine, urine output, and documented duration of dysfunction. CKD required a nephrologist-documented diagnosis and evidence of chronicity for at least 3 months (6); patients with CKD who subsequently met AKI criteria were classified as having AKI superimposed on CKD. Exclusion criteria were death within 72 hours, BMI > 35 kg/m^2^, extensive burns, toxic epidermal necrolysis, severe sepsis with multiorgan dysfunction, or active maintenance dialysis, intermittent hemodialysis, or continuous renal replacement therapy during medication assessment. Of 185 screened records, 80 were included; criterion-specific exclusion counts were not retained.

### 3.3. Sample Size

Cochran's formula for estimating a single proportion under a large-population assumption was applied using Z = 1.96, an absolute precision of d = 0.10, and a planning discordance proportion of p = 0.30, informed by a recent hospital-based study of renal dose adjustment in patients with CKD (9). The value of p = 0.30 was used solely as a preanalysis planning assumption. The calculated sample size was 80.67, corresponding to approximately 81 participants; 80 patients were ultimately included in the study (Figure 1). No finite-population correction was applied.

**Figure 1.**
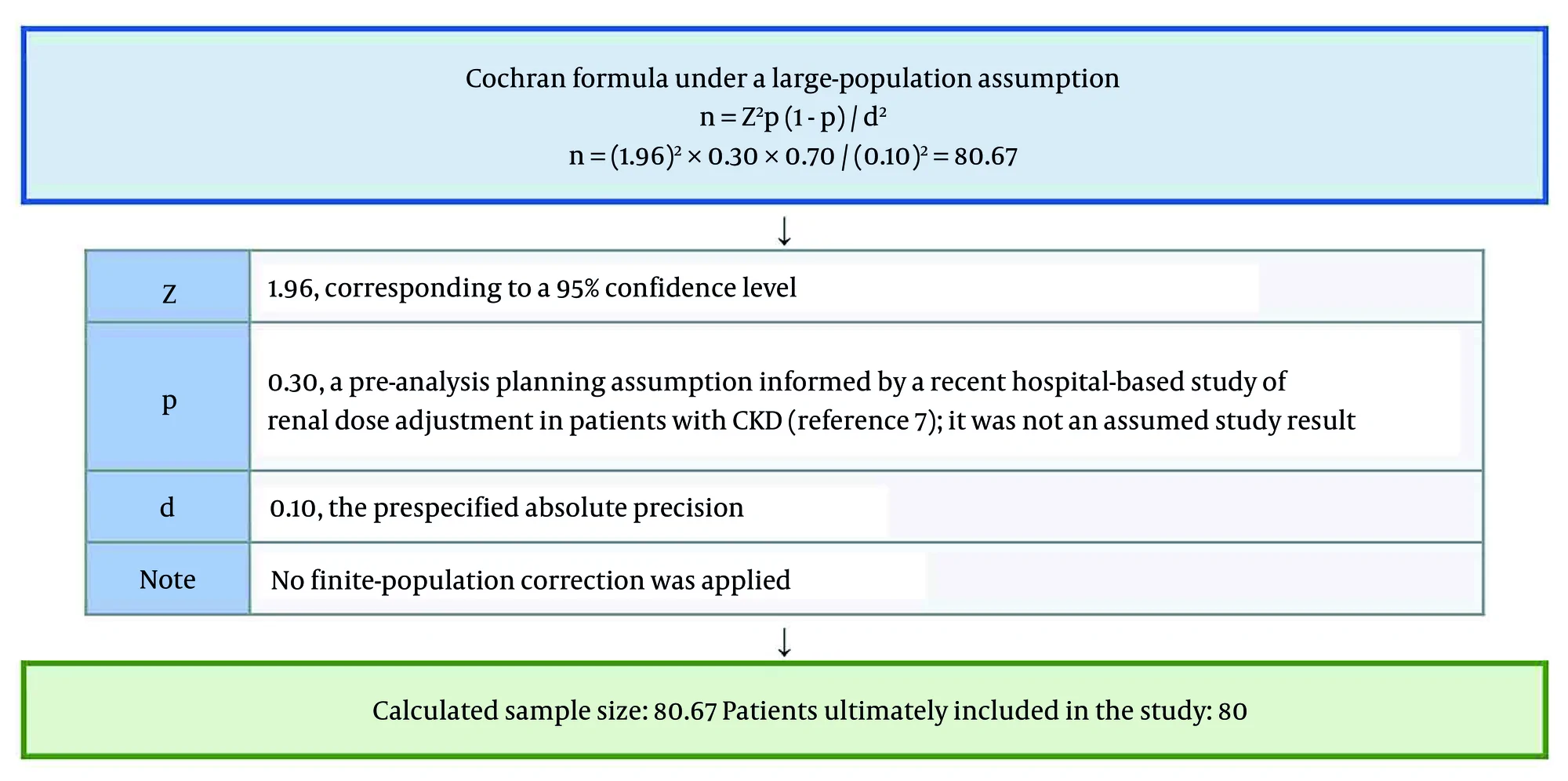
Cochran Sample-Size Calculation for a Single Proportion.

### 3.4. Data Collection and Kidney-Function Assessment

A structured checklist captured demographics, medical history, medications, dose changes, clinical events, laboratory values, and diagnoses from medical records and the hospital information system. Kidney status was determined from serial creatinine values, urine-output records, prior documentation, nephrology assessment, and ultrasonography when available; ultrasonography supported the assessment of obstruction or chronic structural disease but did not independently diagnose AKI. Investigators did not calculate estimated glomerular filtration rate (eGFR) or assign KDIGO G categories. Cockcroft-Gault creatinine clearance (CrCl) was calculated from age, sex, serum creatinine, and chart-recorded body weight (Figure 2); the record did not consistently distinguish measured from estimated ICU weight. Assessment day 1 was the first day on which eligibility and medication assessments were established, and day 7 was the seventh day thereafter. Available intervening creatinine values, urine-output data, medication orders, and the clinical course were reviewed.

**Figure 2.**
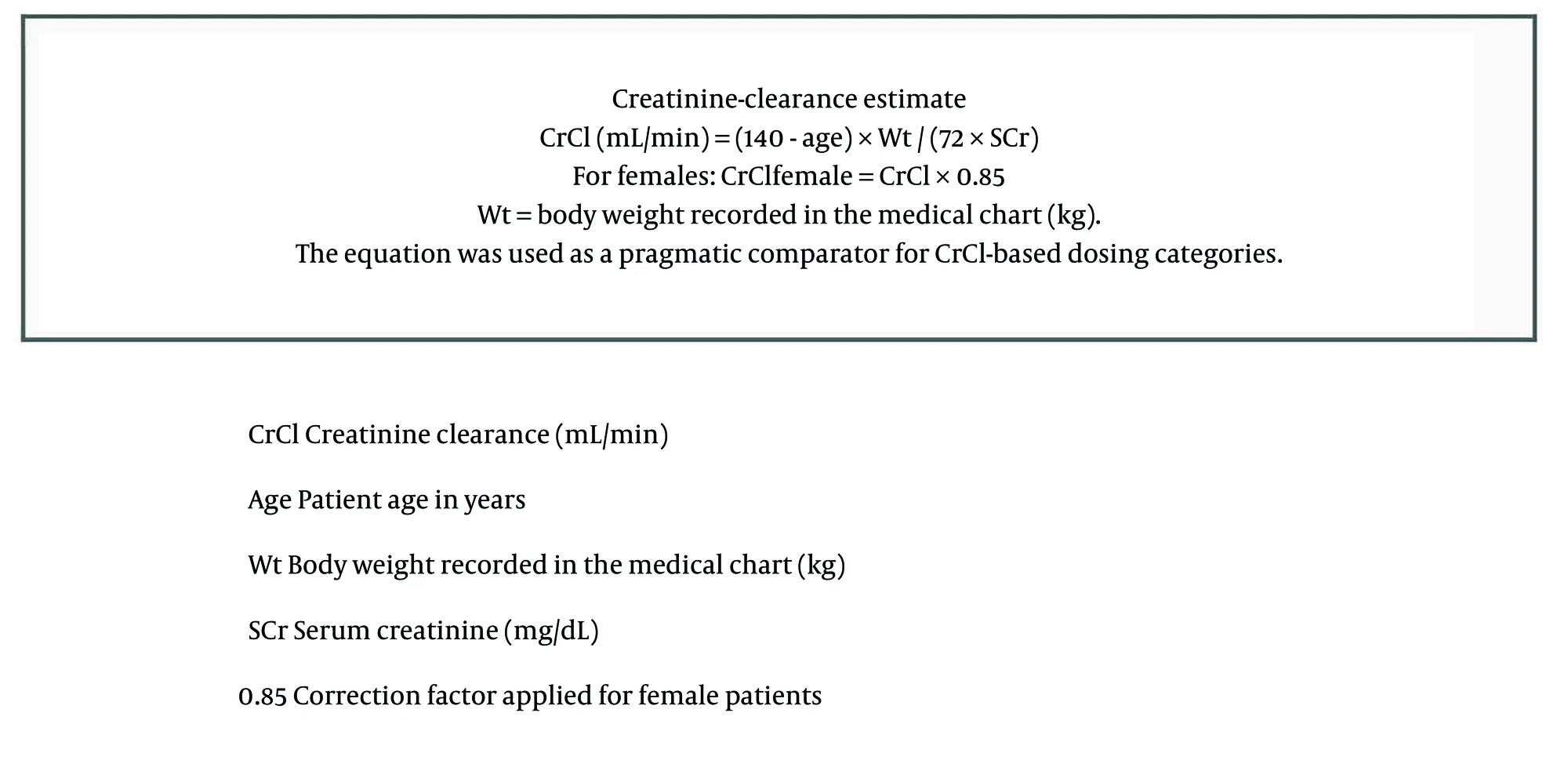
Cockcroft-Gault Equation Used to Estimate Creatinine Clearance. Wt is body weight recorded in the medical chart; 0.85 was applied for females.

### 3.5. Renal Dose-Adjustment Assessment

UpToDate was the prespecified primary dosing reference, and the Sanford Guide was used as a supplementary source, particularly for anti-infectives. When recommendations differed, UpToDate governed classification. Route-specific and physician-documented indication-specific recommendations, including prophylactic versus therapeutic regimens, were applied when relevant. Each patient-medication pair contributed one final classification; a drug present at both assessment points was counted once. Only maintenance-dose amount and interval were assessed, and loading doses were excluded. The evaluated regimen was matched to the most recent renal assessment preceding or contemporaneous with prescribing; unclear temporal relationships were not classified. A regimen was classified as concordant when the dose amount and interval fell within an accepted primary-reference option for the applicable renal category. Lower-than-recommended and higher-than-recommended regimens represented lower or higher total daily maintenance doses resulting from the dose amount, dosing interval, or both, with route considered when specified. In AKI and AKI superimposed on CKD, CrCl served only as a pragmatic comparator for published categories. Creatinine trajectory and available urine output were reviewed, and discordance was not interpreted as confirmed inappropriate exposure. The primary investigator classified regimens; uncertain cases were reviewed with the supervising clinical pharmacy team and resolved by consensus. No drug concentrations, vancomycin area-under-the-curve (AUC) estimates, or other therapeutic drug-monitoring data were available.

### 3.6. Statistical Analysis

SPSS version 25 was used. Continuous variables are reported as mean ± standard deviation or median (interquartile range), and categorical variables as n (%). Paired day-1 and day-7 kidney-function values were compared using paired t tests or Wilcoxon signed-rank tests, as appropriate. The patient was the unit for demographic, phenotype, and prescribing-service summaries; the patient-medication pair was the unit for medication-specific descriptions, with each pair counted once. Drug-specific discordance proportions are presented with Wilson 95% confidence intervals (CIs). The overall discordance proportion was accompanied by a patient-level cluster-bootstrap 95% CI based on 20,000 resamples; resampling patients preserved within-patient clustering. Pair-level inferential tests were not performed. Patient-level categorical comparisons used chi-square or Fisher exact tests. Multivariable models were not fitted because the cohort and several strata were sparse, and no stable model was prespecified. Two-sided P < 0.05 was considered statistically significant.

## 4. Results

Of 185 records assessed, 105 were excluded, and 80 adults were included in the analysis (Figure 3). The mean age was 64.7 ± 16.6 years, and 49 participants (61.3%) were male. The mean chart-recorded weight was 76.8 ± 8.4 kg, and the mean BMI was 25.9 ± 2.3 kg/m^2^. The mean CrCl was 33.8 ± 19.5 mL/min on assessment day 1 and 31.9 ± 14.9 mL/min on day 7 (Table 1). No included patient received active dialysis or continuous renal replacement therapy during medication assessment.

**Table 1.** Demographic and Kidney-Function Characteristics ^a^

Characteristic	Values
**Number of patients**	80
**Age, y**	64.7 ± 16.6
**Male, n (%)**	49 (61.3)
**Height, cm**	172.3 ± 7.9
**Weight, kg**	76.8 ± 8.4
**BMI, kg/m^2^**	25.9 ± 2.3
**Serum creatinine, assessment day 1, mg/dL**	2.7 ± 1.5
**Serum creatinine, assessment day 7, mg/dL**	2.8 ± 1.3

^a^ Values are mean ± SD unless otherwise indicated. Abbreviations: BMI, body mass index; SD, standard deviation.

**Figure 3.**
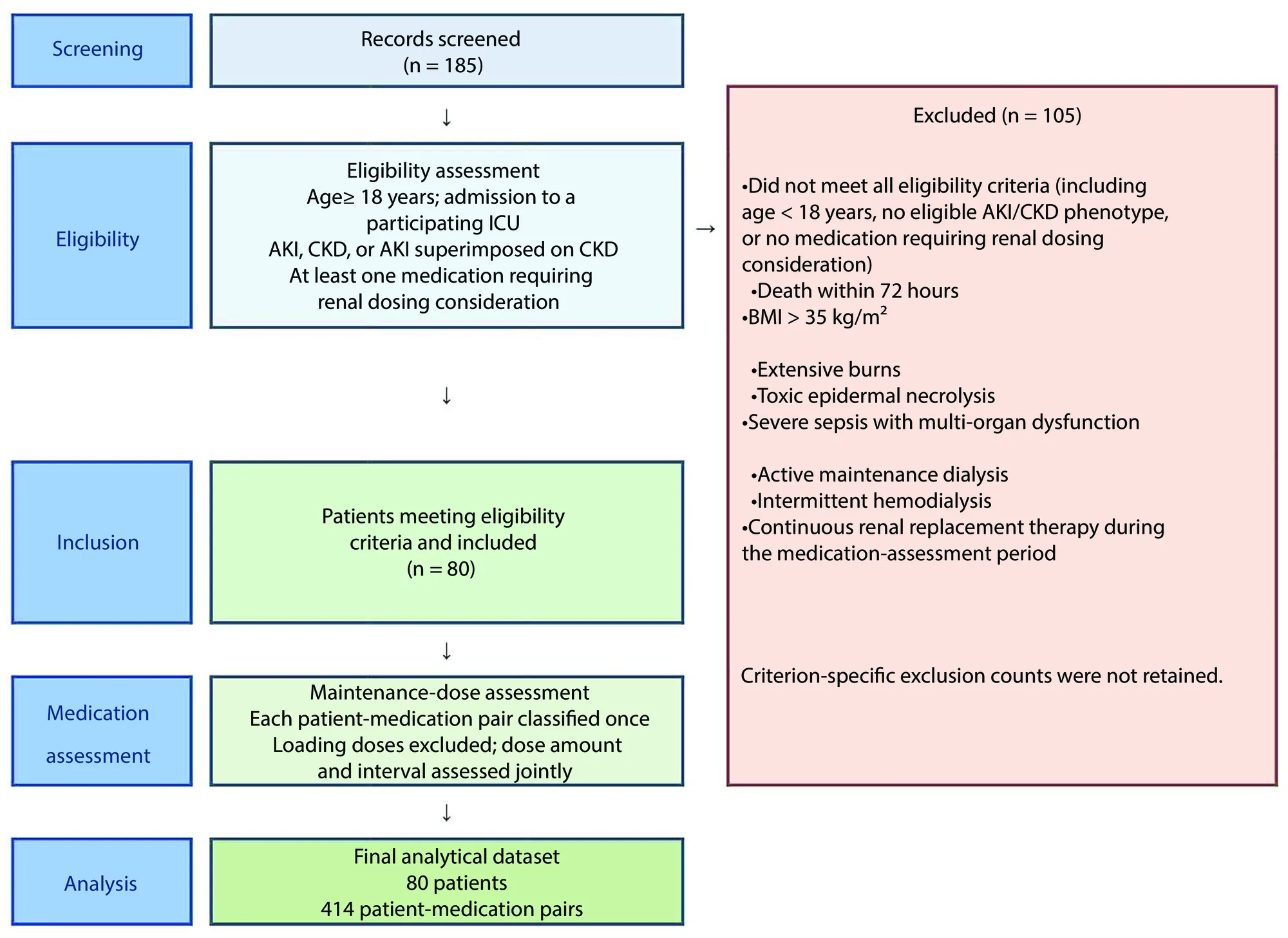
Flow Diagram of Patient Screening and Study Inclusion

AKI superimposed on CKD was present in 38 patients (47.5%), CKD alone in 25 (31.3%), and AKI alone in 17 (21.3%) (Table 2). Among 55 patients classified using RIFLE, 9 (16.4%) were classified as Risk, 20 (36.4%) as Injury, and 26 (47.3%) as Failure. No included patient was classified in the Loss or ESRD outcome categories. The most frequent final diagnoses were pneumonia (13/80, 16.3%), chronic obstructive pulmonary disease (10/80, 12.5%), stroke (6/80, 7.5%), seizure (6/80, 7.5%), and subarachnoid hemorrhage (5/80, 6.3%). These diagnoses described the case mix and were not used to adjudicate antimicrobial indication or infection severity.

**Table 2.** RIFLE Classification and Kidney-Dysfunction Phenotypes ^a^

Classification	No. (%)
**RIFLE classification among patients with AKI or AKI superimposed on CKD (n = 55)**	
Risk	9 (16.4)
Injury	20 (36.4)
Failure	26 (47.3)
Loss	0 (0.0)
ESRD	0 (0.0)
**Kidney-dysfunction phenotype**	
AKI superimposed on CKD	38 (47.5)
CKD alone	25 (31.3)
AKI alone	17 (21.3)

^a^ RIFLE categories used serial creatinine, urine output, and documented duration. Loss and ESRD are outcome categories rather than acute severity stages. Patients with AKI superimposed on CKD were included in the RIFLE denominator. Abbreviations: AKI, acute kidney injury; CKD, chronic kidney disease; ESRD, end-stage renal disease; RIFLE, Risk, Injury, Failure, Loss, and ESRD.

In AKI, the mean serum creatinine decreased from 2.66 ± 1.19 to 2.13 ± 0.68 mg/dL (P = 0.063). In CKD, serum creatinine increased from 3.23 ± 2.11 to 3.93 ± 1.55 mg/dL (P = 0.028); however, this short-term finding was not interpreted as CKD progression because acute illness and treatment could influence creatinine. No statistically significant change was detected in AKI superimposed on CKD.

Among 414 patient-medication pairs, 211 (51.0%; patient-level cluster-bootstrap 95% CI, 46.2%-55.8%) were discordant with the predefined recommendations: 125 (30.2%) were lower and 86 (20.8%) were higher than recommended, whereas 203 (49.0%) were concordant. The confidence interval was estimated by resampling patients to preserve within-patient clustering. Vancomycin had 12/29 discordant regimens (41.4%; 95% CI, 25.5%-59.3%), ciprofloxacin 8/20 (40.0%; 21.9%-61.3%), levofloxacin 6/15 (40.0%; 19.8%-64.3%), meropenem 5/15 (33.3%; 15.2%-58.3%), and levetiracetam 8/27 (29.6%; 15.9%-48.5%) (Table 3). Lower-than-recommended dosing predominated for vancomycin, ciprofloxacin, levofloxacin, and levetiracetam, whereas higher-than-recommended dosing predominated for meropenem.

**Table 3.** Selected Medication-Specific Concordance with Reference-Based Renal Dose-Adjustment Recommendations ^a^

Rank	Drug	Patients	Lower	Concordant	Higher	Total Discordance (%)	95% CI for Total Discordance (%)
**1**	Vancomycin	29	9 (31.0)	17 (58.6)	3 (10.3)	41.4	25.5 - 59.3
**2**	Ciprofloxacin	20	6 (30.0)	12 (60.0)	2 (10.0)	40.0	21.9 - 61.3
**3**	Levofloxacin	15	5 (33.3)	9 (60.0)	1 (6.7)	40.0	19.8 - 64.3
**4**	Meropenem	15	1 (6.7)	10 (66.7)	4 (26.7)	33.3	15.2 - 58.3
**5**	Imipenem	25	4 (16.0)	17 (68.0)	4 (16.0)	32.0	17.2 - 51.6
**6**	Levetiracetam	27	7 (25.9)	19 (70.4)	1 (3.7)	29.6	15.9 - 48.5
**7**	Metformin	22	6 (27.3)	16 (72.7)	0 (0.0)	27.3	13.2 - 48.2
**8**	Ampicillin-sulbactam	13	2 (15.4)	10 (76.9)	1 (7.7)	23.1	8.2 - 50.3
**9**	Colistin	18	4 (22.2)	14 (77.8)	0 (0.0)	22.2	9.0 - 45.2
**10**	Ceftazidime	19	2 (10.5)	15 (78.9)	2 (10.5)	21.1	8.5 - 43.3
**11**	Amikacin	5	0 (0.0)	4 (80.0)	1 (20.0)	20.0	3.6 - 62.4
**12**	Gabapentin	18	3 (16.7)	15 (83.3)	0 (0.0)	16.7	5.8 - 39.2
**13**	Cotrimoxazole	18	1 (5.6)	15 (83.3)	2 (11.1)	16.7	5.8 - 39.2

^a^ Values are expressed as No (%), (95% CI) or Number unless otherwise indicated. Thirteen selected medications are shown. Each drug contributed at most one observation per patient. Loading doses were excluded, and physician-documented indication-specific recommendations were used when applicable. Wilson 95% CIs are shown.

At the patient level, the nephrology service had the highest proportion of patients with at least one discordant regimen (5/8, 62.5%), followed by pulmonology (8/13, 61.5%) and general internal medicine (3/5, 60.0%) (Table 4). These unadjusted proportions describe the service-level case mix and should not be interpreted as comparisons of individual physician performance.

**Table 4.** Renal Dosing Discordance According to Prescribing Service ^a^

Rank	Prescribing Service	Patients	Patients with ≥ 1 Discordant Regimen	Medication Most Often Associated with Discordance
**1**	Nephrology	8 (10.0)	5/8 (62.5)	Colistin
**2**	Pulmonology	13 (16.3)	8/13 (61.5)	Ciprofloxacin
**3**	General internal medicine	5 (6.3)	3/5 (60.0)	Meropenem
**4**	Neurology	24 (30.0)	12/24 (50.0)	Levetiracetam
**5**	Rheumatology	4 (5.0)	2/4 (50.0)	Allopurinol
**6**	Endocrinology	4 (5.0)	2/4 (50.0)	Metformin
**7**	General surgery	2 (2.5)	1/2 (50.0)	Ceftazidime
**8**	Infectious diseases	14 (17.5)	6/14 (42.9)	Vancomycin
**9**	Anesthesiology	3 (3.8)	1/3 (33.3)	Meropenem
**10**	Other specialties	3 (3.8)	1/3 (33.3)	Colistin

^a^ Values are expressed as n (%) or n/N (%). Percentages in the third column use the total cohort (N = 80); percentages in the fourth use patients within each service. These unadjusted proportions describe service-level case mix and do not compare individual physicians.

## 5. Discussion

This study identified frequent discordance between prescribed maintenance regimens and predefined renal dosing references across AKI, CKD, and AKI superimposed on CKD. Its principal contribution is a reproducible reference-concordance assessment that defines dose amount, interval, route, timing, and reference hierarchy while avoiding the assumption that a table-based discrepancy implies inappropriate exposure. The overall pair-level discordance proportion was 51.0%, reflecting the descriptive burden among the evaluated regimens rather than a patient-level risk estimate, because multiple medications could be associated with the same patient.

The observed frequency is consistent with the substantial variation in renal dosing reported in hospitalized CKD populations, although direct numerical comparison is difficult because studies differ in renal definitions, medication lists, reference sources, and units of analysis (9, 10). These differences are especially important in the ICU. Studies that classify every prescription line as independent may produce narrow confidence intervals despite within-patient correlation, whereas patient-level summaries may conceal repeated discordant regimens. We therefore report medication-specific Wilson intervals, for which each patient contributes at most once per drug, while treating the overall pair-level proportion descriptively.

The pair-level estimate should be interpreted in relation to the study design. Previous work has documented a substantial burden of renal dose-adjustment problems among hospitalized patients with CKD, supporting the planning assumption used for sample-size estimation (9). However, differences in patient characteristics, medication profiles, clinical settings, and outcome definitions limit direct comparison with the present ICU cohort.

The distinction between stable CKD and dynamic AKI is central to interpretation. In CKD, a stable creatinine-based estimate is more likely to approximate the renal categories used in standard drug references. During AKI, however, serum creatinine is delayed and non-steady-state; 2 patients with the same contemporaneous Cockcroft-Gault estimate may have different true drug clearance if one is deteriorating and the other is recovering (2, 7, 8, 11). Serial creatinine, available urine output, and medication timing were reviewed, but these measures cannot replace measured creatinine clearance, kinetic eGFR, cystatin C-based assessment, pharmacokinetic modeling, or therapeutic drug monitoring. Accordingly, “discordance” is more appropriate than “error” for AKI and AKI superimposed on CKD.

Even among patients with CKD, acute illness, altered muscle mass, nutritional status, and fluid shifts may influence serum creatinine independently of glomerular filtration. The use of chart-recorded rather than ideal body weight reflects the information available to clinicians at the time of prescribing. This analysis reduces, but does not eliminate, uncertainty related to weight estimation and volume status in the ICU setting (12).

Discordance was concentrated in anti-infective and neurologic medications requiring individualized interpretation of dose, interval, renal trajectory, infection site, organism susceptibility, and sometimes therapeutic drug monitoring. Among the selected medications shown in Table 3, vancomycin had the highest discordance proportion, predominantly due to lower-than-recommended regimens. Current guidance favors exposure-guided monitoring for serious infections, commonly using an AUC/minimum inhibitory concentration target of 400 - 600 mg·h/L (13). No concentrations or AUC estimates were available, so the study cannot determine whether reference discordance resulted in subtherapeutic or toxic exposure. This absence also prevents validation of Cockcroft-Gault-based categorization against a clinically relevant exposure standard, particularly in conditions such as augmented renal clearance, in which standard equations often underestimate actual drug clearance (14).

Fluoroquinolone regimens were also frequently lower than the reference recommendations. In critical illness, impaired and augmented renal clearance may both occur, while serum creatinine can lag behind actual changes in filtration (1, 2, 8). A lower reference-based regimen therefore cannot be equated with microbiological underexposure without organism identification, minimum inhibitory concentration, measured drug concentrations, and outcome data.

Meropenem more often showed higher-than-recommended dosing. Accumulation may increase the risk of neurotoxicity in severe kidney impairment, whereas aggressive or prolonged-infusion regimens may be selected intentionally to achieve pharmacodynamic targets. The present design could not distinguish individualized dosing from unintended excess exposure (2, 8).

Levetiracetam showed predominantly lower-than-recommended regimens. Its clearance is closely related to kidney function, but critical illness, altered volume of distribution, augmented clearance, seizure severity, and concomitant therapy may influence dose selection (15). No concentration or seizure-control outcome validated the reference classification. For drugs with distinct prophylactic and therapeutic regimens, the applicable reference was selected according to physician-documented intent; nevertheless, indication and infection severity were not analyzed as cohort-level outcomes.

Service-level proportions were highest in nephrology and pulmonology, but denominators were small and services treated different case mixes. Nephrology may manage more severe or unstable kidney dysfunction, whereas pulmonology may prescribe more renally eliminated antimicrobials. These unadjusted proportions should not be interpreted as rankings of physician performance. Similarly, lower- and higher-than-recommended classifications indicate the direction of difference from a reference, not the direction of clinical harm. Lower exposure may compromise efficacy, whereas higher exposure may increase accumulation and toxicity, but the preferred regimen also depends on clinical context.

The distinction between lower- and higher-than-recommended dosing is clinically relevant but is not, by itself, directional evidence of harm. Lower exposure may jeopardize efficacy for time- or concentration-dependent antimicrobials, whereas higher exposure may increase accumulation and toxicity. However, infection severity, site, organism susceptibility, residual renal function, expected renal recovery, and treatment goals may justify a regimen that differs from a general reference table. Because these variables were incompletely captured, the present results are best used to identify prescriptions requiring review rather than to retrospectively judge prescribing decisions.

From a medication-safety perspective, a discordant regimen should prompt targeted review of the current renal trajectory, indication, route, dose, interval, and monitoring rather than automatic correction. This approach can reduce unnecessary changes when individualized dosing is justified while directing attention to regimens requiring clarification. Computerized decision support and clinical pharmacy review may improve medication-use processes, but effectiveness must be evaluated prospectively in the local setting (16). Future studies should prespecify patient-level clustering, model time-varying renal function and dosing, include measured or kinetic renal estimates, and link discordance to drug exposure and clinical outcomes. Prospective implementation should also record the prescriber’s rationale, whether a clinical pharmacist recommendation was accepted, whether the regimen changed, and whether that change affected drug exposure, safety, effectiveness, or length of stay.

This study should be interpreted in light of several limitations. Its single-center design, modest sample size, and 10% absolute precision may limit generalizability. Exclusion of highly unstable patients and those receiving active renal replacement therapy reduced representation of the most complex dosing scenarios, and criterion-specific exclusion counts were unavailable. Cockcroft-Gault estimates may be less reliable in non-steady-state AKI, while measured renal clearance, cystatin C, kinetic estimates, therapeutic drug monitoring, infection-severity measures, and clinical outcomes were unavailable. Each patient-medication pair was classified once, so time-varying changes were not modeled. Because the study relied on routine clinical records, prescribing rationale and some relevant clinical variables were not consistently documented. In addition, the cross-sectional design did not evaluate a pharmacist-led intervention and cannot determine whether such an intervention would reduce dosing discordance. Nevertheless, the predefined reference hierarchy, review of serial renal data, and explicit classification criteria support the internal consistency of the descriptive findings.

In conclusion, reference-based renal dosing discordance was common, particularly for selected antimicrobials. These descriptive findings warrant prospective validation using dynamic renal measures, exposure monitoring where appropriate, and patient-centered outcomes. They support formal evaluation, rather than presumption, of structured clinical pharmacy and renal-dosing decision support in critically ill patients.

## Data Availability

The dataset is not publicly available because it contains clinical information from critically ill patients. De-identified data may be available from the corresponding author on reasonable scientific request and subject to ethical and institutional approval.

## Clinical Trial Registration Code:

No
